# Maize (*Zea mays* L.) genome size indicated by 180-bp knob abundance is associated with flowering time

**DOI:** 10.1038/s41598-017-06153-8

**Published:** 2017-07-20

**Authors:** Yinqiao Jian, Cheng Xu, Zifeng Guo, Shanhong Wang, Yunbi Xu, Cheng Zou

**Affiliations:** 1 Institute of Crop Science, National Key Facility of Crop Gene Resources and Genetic Improvement, Chinese Academy of Agricultural Sciences, Beijing, 100081 China; 2 International Maize and Wheat Improvement Center (CIMMYT), El Batán, Texcoco, 56130 Mexico

## Abstract

Flowering time is considered one of the most important agronomic traits in maize (*Zea mays* L.), and previous studies have indicated that this trait is correlated with genome size. We observed a significant difference in genome size between tropical and temperate inbred lines and a moderate positive correlation between genome size and 180-bp knob abundance determined by high-throughput sequencing in maize inbred lines in this study. We assembled the reads that were mapped to 180-bp knob sequences and found that the top ten abundant 180-bp knob sequences are highly variable. Moreover, our results indicate that genome size is associated with the flowering time of both male and female flowers, in both tropical and temperate inbred lines and under both tropical and temperate environments. To identify loci associated with genome size, we performed a genome-wide association study. The analysis identified three genomic regions associated with genome size, of which two were novel while the third one is located close to the known knobs K8L1 and K8L2. Overall, our results indicate that selection for breeding materials with earlier flowering times can be assisted by choosing germplasms with smaller genome sizes and that genome size can be determined based on the abundance of 180-bp knobs.

## Introduction

Maize originated from the lowland tropics in South America and is now grown almost all over the world from latitudes 40**°**S to 58**°**N (https://www.worldcornproduction.com). Behind this wide distribution is extreme diversity both in phenotype^1–3^ and genotype^4–9^. In addition to phenotypic and genetic variation, large variation in genome size has been observed among tropical and temperate maize germplasm^10^, including differences in knob content^5^. Genome size in the *Zea* genus varies within species as well as between species^11^ and is correlated with a wide range of phenotypes, such as seed mass^12^, leaf size^13^, growth rate^14^ and flowering time^15^. The B73 reference genome size is 5.64 pg/2C^16^, and there is at least a 30% difference in genome size among maize inbred lines^5^. Most of this variation is caused by differences in the amount of repetitive sequence, and the proportion of unique regions does not vary significantly among different lines^17^. Alterations in the proportion of repetitive sequence have been caused by temperature differences at different geographic locations, longitudes, and latitudes^16^.

The maize genome is composed of 85% repetitive sequence^18^, 9.4% of which is found in knobs^19, 20^. Knobs were first discovered by McClintock^21^ and can provide evidence for the physical exchange of chromosomal segments during crossing over^22^. These knobs are the major components of heterochromatic regions and consist of 350-bp and 180-bp repeating units^16, 18, 23^. Previous studies suggest that knob number is correlated with the amount of nuclear DNA among different maize varieties^4^, and knob size is correlated with the amount of 180-bp repeat sequence observed by *in situ* hybridization^24^. In an oat-maize chromosome alien addition line, the 180-bp knob is interrupted by retrotransposable elements, and these elements constitute about 30% of the sequence in knob DNA regions of chromosome 9^25^. High levels of polymorphism in knob size and number have also been found among different maize strains^20^. Different maize inbred lines showed highly variable knob numbers, ranging from zero^25^ to 20 or even more, which are located in one or more chromosomes^26^. The abundance of 180-bp knobs has been investigated using resequencing data^5, 23^; however, a relatively small number of inbred lines were used in those studies.

Maize exhibits huge variation in flowering time, with days to flowering ranging from 35 to 120 days^27^. So far, most studies have focused on the genes regulating flowering time, such as *Vgt1* ^28^, *ZCN8* ^29^, *id1* ^30^ and *ZmCCT* ^31, 32^. Many studies have also reported a large number of quantitative trait loci (QTL)^33^ for flowering time, each with small phenotypic effects^34^. However, less effort has been made to study the relationship between genome size and knobs on flowering time. For example, six cycles of selection in maize for early flowering has been reported to decrease genome size^15^. Researchers have hypothesized that maize lines with larger genome sizes need more time to complete vegetative growth^14^, which may explain the link between flowering time and genome size.

One approach that has been successfully used to identify the genetic basis of quantitative traits is genome-wide association study (GWAS), which is a complementary strategy for linkage analysis. GWAS is a useful approach to dissect complex agronomic traits in maize for quick linkage disequilibrium (LD) analysis. With the availability of low-cost and high-throughput single nucleotide polymorphism (SNP) genotyping platforms, high-throughput microarray and sequencing technologies, GWAS has been successfully applied in maize to identify major effect genes and genomic regions associated with several traits including oil biosynthesis in kernels^35^, leaf architecture^36^, *ZmDREB* and *ZmNAC111* that increase drought tolerance at seedling stage^37, 38^, *crtRB1* that increases grain β-carotene concentration^39^ and other QTL in the nested association mapping (NAM) population^34^.

In the present study, knob abundance in different maize inbred lines was calculated and we investigated both male and female flowering time in diverse tropical and temperate maize inbred lines and its relationship to genome size in both temperate and tropical regions. To identify loci associated with genome size, we also performed a genome-wide association study.

## Results

### Genome size in tropical and temperate inbred lines

**Figure 1:**
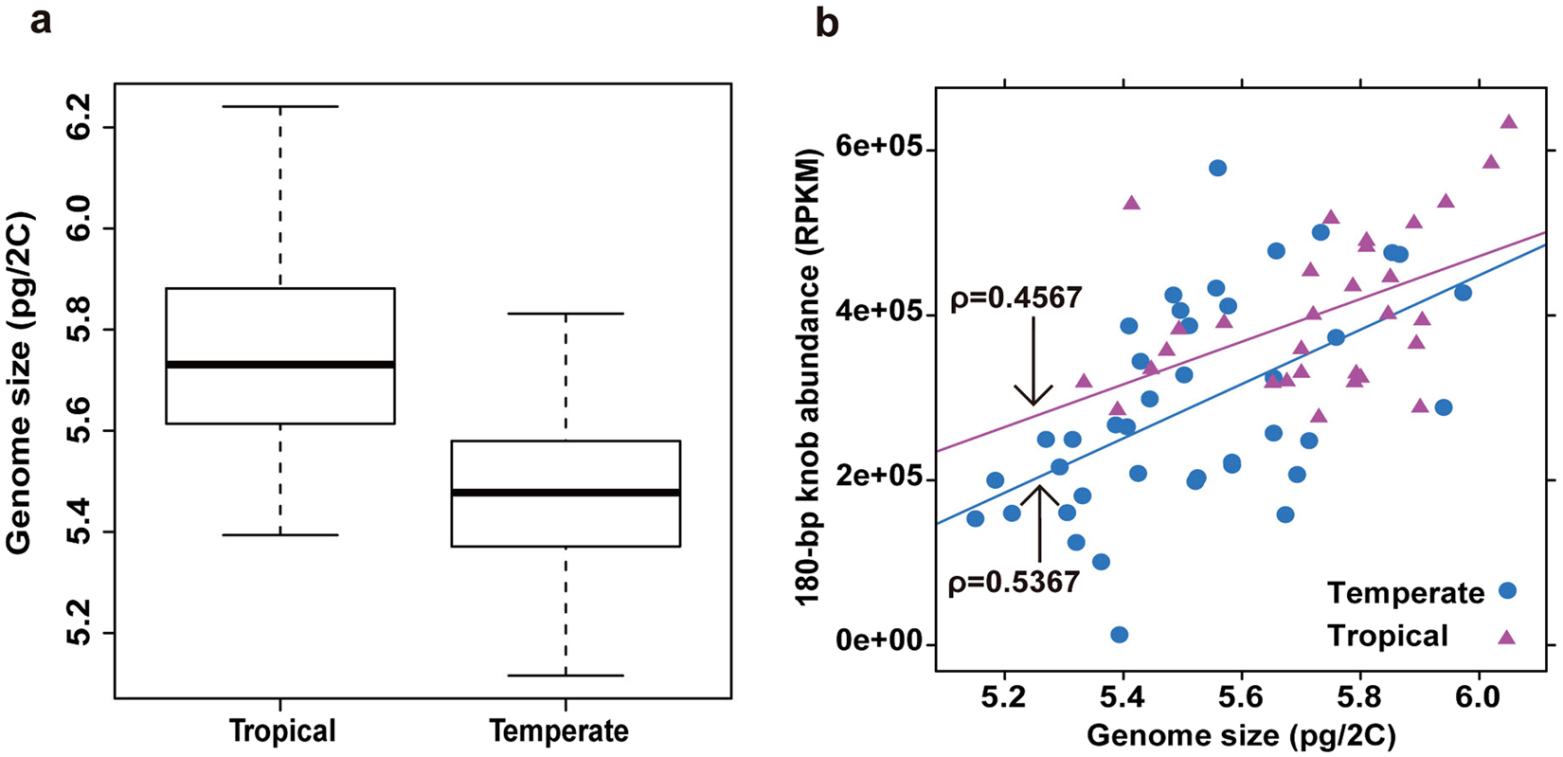
(**a**) Significant difference in genome size (pg) between tropical (n = 74) and temperate (n = 74) inbred lines (*P* = 2e-16 based on one-way ANOVA). The boxes indicate the first quartile (bottom line), the median (central line) and the third quantile (top line). The whiskers represent the standard deviation. (**b**) Correlation between genome size and RPKM of 180-bp knob based on genome size data from 70 genomes. The x-axis is absolute nuclear DNA content, and the y-axis is RPKM, which is a measure of 180-bp knob abundance. Blue circles and purple triangles indicate temperate and tropical maize lines, respectively. Regression lines are shown for both the temperate (blue circle) and tropical (purple triangle) lines. Correlation values are ρ = 0.5367 for temperate inbreds and ρ = 0.4567 for tropical inbreds.

To test if there was a significant difference in genome size between tropical and temperate maize inbred lines, the genome sizes of different maize ecotypes were determined by flow cytometry. Based on ANOVA, the genome size of tropical maize inbred lines was significantly (*P* < 0.05) higher than the temperate inbred lines (Fig. 1a). The genome size of tropical inbreds ranged from 5.39 to 6.24 pg, with an average of 5.75 pg and a coefficient of variation (CV) of 0.03, while those of the temperate inbreds ranged between 5.12 and 5.83 pg with an average of 5.48 pg and a CV of 0.03 (Fig. 1a, Table S1).

### Correlation between genome size and knob abundance

In order to gain an insight on the relationship between 180-bp knob abundance and genome size in tropical and temperate ecotypes, DNA libraries prepared from 70 maize inbred lines originate from a wide range of latitudes were sequenced, and knob abundance was determined based on the number of reads that mapped to 180-bp knob sequences (Reads Per Kilobase of region per Million mapped reads; RPKM). Abundance of 180-bp knob sequences was highly variable, ranging from 11,854 for the temperate inbred line Ji853 to 632,730 for the tropical inbred line CML511 (Table S2). The overall average 180-bp knob abundance is 338,455 with a coefficient of variation of 0.38. The low knob abundance and small genome size of Ji853 prompted us to test if there was a correlation between 180-bp knob abundance and genome size. Spearman’s correlation showed that there is a highly significant positive correlation between genome size and knob abundance (Fig. 1b; ρ = 0.5367, *P* < 0.01 for temperate inbreds and ρ = 0.4567, *P* = 0.01 for tropical inbreds). Ignoring ecotype, the value of ρ for the correlation between genome size and 180-bp knob abundance is 0.6056 (*P* < 0.01).

### 180-bp knobs are highly variable

**Figure 2:**
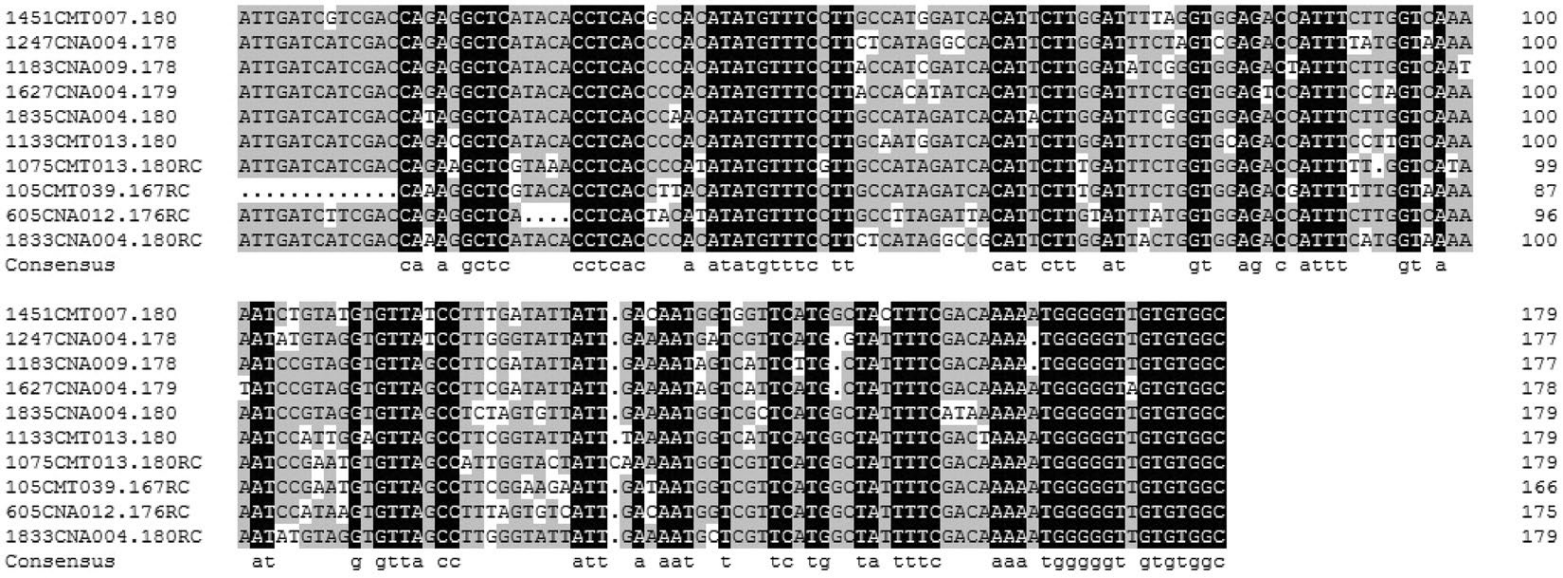
180-bp knob sequences are highly variable. To determine the diversity of 180-bp knob sequences, the top ten fasta abundant sequences were aligned by DNAMAN 7.0. Sequence names reflect the assembled sequence number, the name of the inbred line, and the length of the knob. For example, 1833CNA004.180 is the 1833rd sequence of the inbred line CNA004 and is 180-bp long. The number to the right of each sequence indicates the length of the sequence. The last line shows the 180-bp knob consensus sequence.

In order use 180-bp knob abundance as a marker to estimate genome size, we determined whether 180-bp knob sequences are monomorphic or polymorphic among different germplasm. Because the dataset was too large to assemble 180-bp knob sequences for all inbred lines, we used a subset of 16 of the 70 inbred lines that showed large differences in genome size. Paired-end sequencing reads from 16 inbred lines were aligned to 180-bp knob sequences download from NCBI. The mapped reads were used to assemble line-specific 180-bp knob sequence. We obtained 11,829 180-bp knob sequences with a different number of contigs for each line (Table 1). Reads from the 16 resequenced lines were further mapped to these 11,829 knob sequences. The differences in the numbers of reads mapped to the 180-bp knobs among the 16 inbred lines indicated that 180-bp knob abundance is highly variable (Table S3). The top ten abundant knob sequences shared about 50% similarity (Fig. 2), which indicate that if we want to estimate the abundance of 180-bp knob and use it as a marker for estimating genome size, we had to establish a highly variable database. Otherwise, the use of incomplete pool of 180-bp sequences will lead to an incorrect estimation of the genome size.

**Table 1:** The number of reads mapped to 180-bp knobs and the number of assembled 180-bp knob sequences for 16 resequenced maize inbred lines.

Sample No.	Ecotype	No. of mapped reads	No. of assembled 180-bp knobs
CNA004	temperate	5.84E + 05	1100
CNA008	temperate	7.54E + 06	851
CNA012	temperate	9.92E + 06	726
CNA009	temperate	1.17E + 07	951
CMT015	tropical	1.22E + 07	604
CMT007	tropical	1.22E + 07	751
CNA020	temperate	1.35E + 07	453
CMT024	tropical	1.42E + 07	401
CMT020	tropical	1.63E + 07	639
CMT039	tropical	1.70E + 07	789
CMT002	tropical	1.71E + 07	767
CNA045	temperate	1.81E + 07	670
CMT028	tropical	2.00E + 07	709
CMT013	tropical	2.22E + 07	637
CMT008	tropical	2.50E + 07	838
CMT019	tropical	3.22E + 07	943

### Correlation between flowering time and genome size

We first evaluated the flowering time of 85 temperate and 74 tropical maize inbred lines grown at two locations. Using Shapiro-Wilk test, both days to anthesis (DTA) for male flowering and days to silking (DTS) for female flowering showed significant deviation (*P* < 0.008) from normality in both tropical (Hainan) and temperate (Beijing) zones(Supplementary Figure 1). According to the Supplemental Figure S1, both DTA and DTS in the tropical zone shows bimodal distribution, while those in temperate show unimodal distribution. Inbred lines grown in tropical and temperate environments required from 48 to 78 and from 70 to 106 days to flower, respectively (Table S4). Anthesis occurred on average 1.9 and 3.2 days earlier than silking in the tropical and temperate regions, respectively (Table S4).

**Figure 3:**
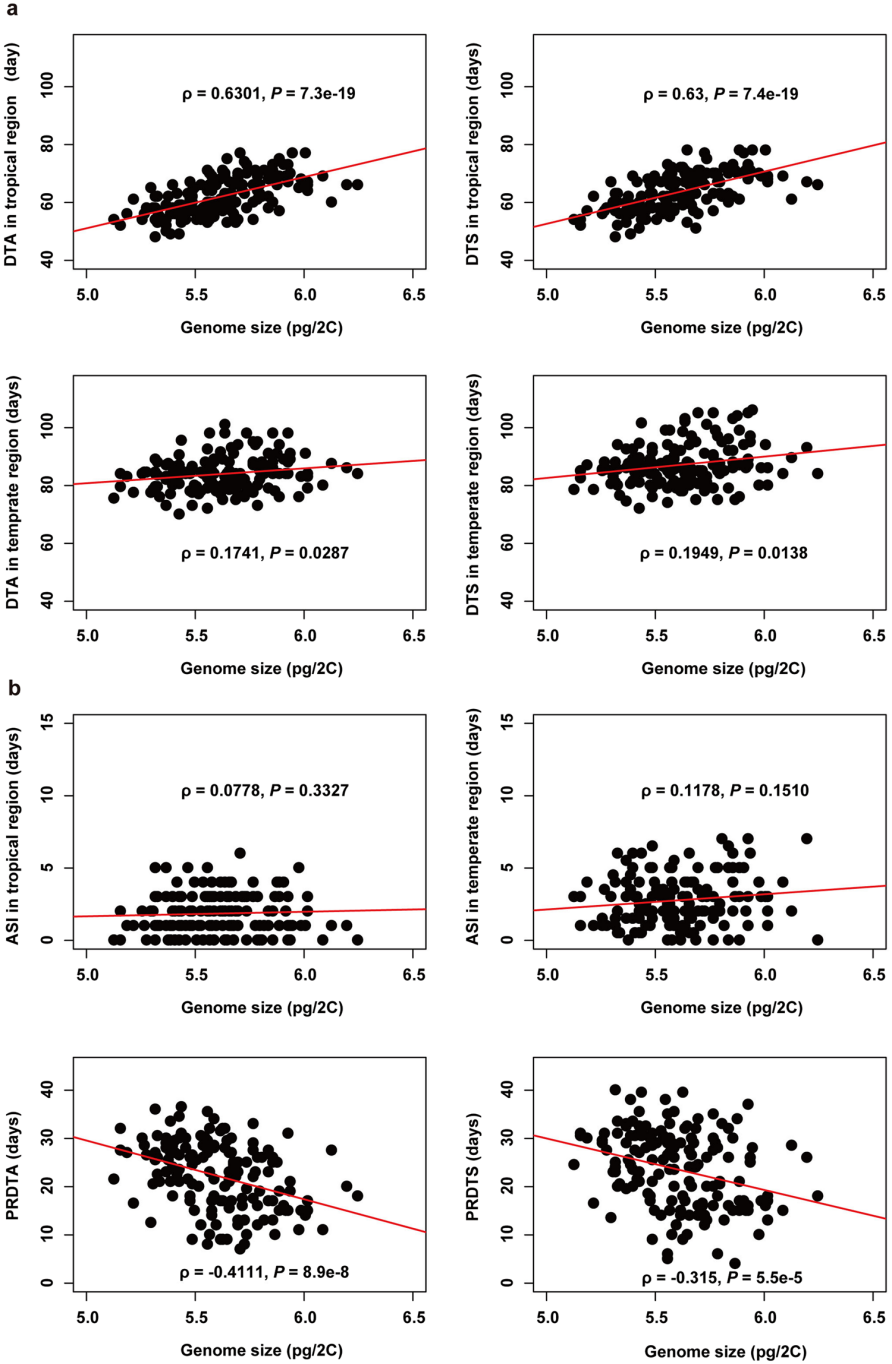
Moderate correlation between flowering time and genome size. (**a**) Plot of genome size and flowering time (DTA and DTS) for maize inbred lines grown in tropical and temperate regions. The x-axis is absolute nuclear content and the y-axis is flowering time. Spearman’s correlation coefficient (ρ) is shown for each plot. (**b**) Relationship between ASI, PRDTA and PRDTS (x-axis) and genome size (y-axis) in tropical and temperate regions. Spearman’s correlation coefficient (ρ) is shown for each plot. ASI in the tropical and temperate regions did not correlate with genome size. Both PRDTA and PRDTS showed a moderate negative correlation with genome size. PR: photoperiod response.

We next determined the relationship between genome size and flowering time. For both tropical and temperate inbred lines, genome size was moderately correlated with male and female flowering time (*P* < 0.05). Regardless of sex, there was higher correlation between genome size and flowering time in tropical inbreds than in temperate inbreds (Fig. 3a). The contribution of genome size to flowering time in tropical inbreds was also larger than in temperate inbreds based on a linear model. When population structure was introduced to the linear model as described by Tenaillon *et al*.^14^, the contribution of genome size to DTA in and DTS in tropical ecotypes became larger than without including population structure (Table 2), indicating that the correlation between genome size and flowering time is independent of population structure. However, when kinship was introduced, the model was no longer significant for all traits (*P* > 0.05). We further analyzed the correlation between genome size and other traits related to flowering time: anthesis-silk interval (ASI), photoperiod response of day to anthesis (PRDTA) and photoperiod response of day to silking (PRDTS) (Table S4). ASI is not correlated with genome size in either the tropical or temperate regions (Fig. 3b). Both PRDTA and PRDTS are moderately negatively correlated with genome size (Fig. 3b and Table S4). Overall, our results indicate that there is a broad association of genome size with flowering time irrespective of sex, ecotype and environment.

**Table 2:** R^2^ (contribution coefficient) and *P* for each contribution coefficient determined from a linear model of genome size.

Data	Items	DTAHN	DTSHN	DTABJ	DTSBJ	ASIHN	ASIBJ	PRDTA	PRDTS
159	R^2^	0.3464	0.3464	0.05	0.055	0.005	0.024	0.136	0.088
	*P*	3.4E-16	3.4E-16	0.005	0.003	0.394	0.053	<0.0001	0.0002
136	R^2^	0.3312	0.3317	0.0156	0.0183	0.0059	0.0124	0.1946	0.1457
	*P*	2.3E-13	2.2E-13	0.147	0.1163	0.3723	0.1975	7.6E-08	<0.0001
136 + PC	R^2^	0.5046	0.5021	0.0229	0.0294	0.0159	0.0270	0.2988	0.2182
	*P*	2.2E-16	2.2E-16	0.2136	0.1439	0.3448	0.1623	5.6E-11	7.8E-08
136 + PC + Kinship	R^2^	0.7820	0.8217	0.4211	0.4033	0.2023	0.8284	0.3179	0.3285
	*P*	0.2950	0.2360	0.2900	0.3240	0.1380	0.4240	0.1840	0.1410

Contribution coefficients for different indicators of flowering time for 159 tropical and temperate maize inbred lines in different zones are shown. A total of 136 out of 159 was fit to a linear model with population structure introduced and further with kinship introduced.

DTS: days to silking; DTA: days to anthesis; ASI: anthesis-silk interval; PRDTA: photoperiod response of day to anthesis; PRDTS: photoperiod response of day to silking; HN: Hainan, represents tropical region; BJ: Beijing, represents temperate region.

### GWAS of genome size

**Figure 4:**
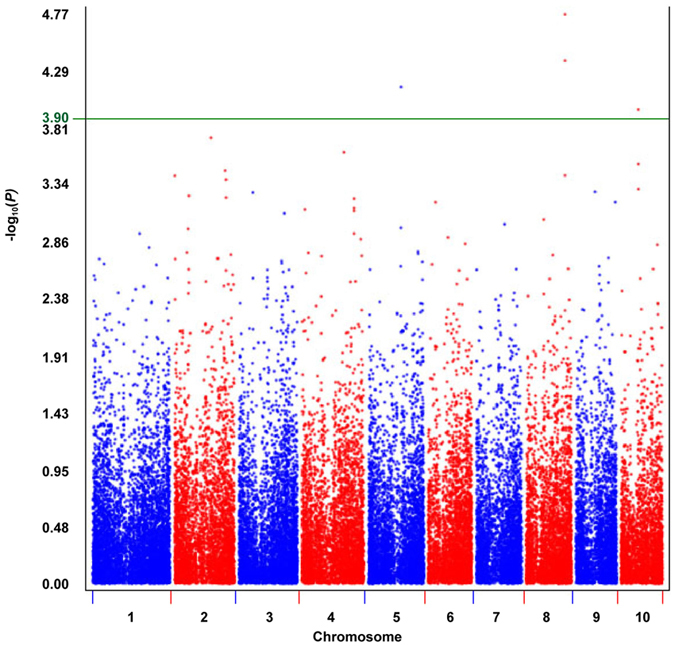
GWAS for genome size identified three genomic regions close to knob clusters. Chromosomes are shown on the x-axis. *P* are shown in −log_10_ scale on the y-axis.

In order to identify genomic regions associated with genome size, we conducted a GWAS in a diverse panel of including 82 temperate and 93 tropical/subtropical inbred lines (Table S5). This panel was genotyped using a recently developed 55 K SNP Chip^40^ and genome size was evaluated by flow cytometry. Using a minimum threshold value of −log_10_ (*P*) = 3.9, we identified three genomic regions that individually explained between 9.2 and 10.8% of the phenotypic variation for flowering time (Fig. 4 and Table 3). Two genomic regions are represented by a single marker that mapped in the centromere on chromosome 5 (bin 5.04) and chromosome 10 (bin 10.03); both regions are novel. The third genomic region is the most significant and is represented by two markers that are located on the long arm of chromosome 8 (bin 8.06), close to the known knobs K8L1 and K8L2 (Table 3).

**Table 3:** Candidate positions related with genome size.

SNP	Chr	Base Pair	*P*	No. of significant SNPs	R^2^	Bin*	Annotation
QTL_1	8	151205865	1.70E-05	2	0.108	8.06	K8L1, K8L2 in maizeGDB
QTL_2	5	128340042	6.92E-05	1	0.098	5.04	Novel
QTL_3	10	62872930	1.07E-04	1	0.092	10.03	Novel

^*^Bin: chromosomal location.

## Discussion

A number of researchers have reported the significance of small-effect QTL in flowering time variation^33, 34, 41^. However, little research has been focused on the influence of genome size on flowering time. Understanding the relationship between flowering time and genome size will aid the selection for early flowering lines in maize breeding. Here, we used a combination of high-throughput sequencing technology and flow cytometry to understand the association between flowering time and genome size indicated by 180-bp knob abundance. The association between absolute DNA content of nuclei indicated by 180-bp knob abundance and flowering time indicates that 180-bp knob abundance can be used in maize breeding.

Knobs have experienced considerable decline due to selection. For example, teosinte which is the ancestor of maize, has twice as many knobs as modern maize lines^5^. Previous efforts have been made to find knob polymorphisms^20, 24, 26, 42^. However, those studies were based on PCR amplification using conserved primer sequences, which enabled discovery of only limited types of knob sequence variation^16^. In the present study, by using a *de novo* assembly method, we found much more polymorphism in knob sequence. We predict that 180-bp knob abundance, as well as 350-bp knobs (known as TR-1)^20^, combined with flowering time genes, contribute to variation in flowering time in maize. Previous researchers have suggested that the increased packaging of heterochromatin caused by a larger number of 180-bp and 350-bp knobs increases the time needed for the DNA synthesis phase and thus cycle time^24^.

Our results showed a moderate correlation between flowering time and genome size, which suggests the possible influence of genome size on flowering time. However, like previously identified small-effect QTL, genome size also does not have a large effect on flowering time. The moderate correlation that we observed is also in agreement with a previous study which concluded that selection for earlier flowering leads to reduced genome size^15^. In addition to flowering time, growth rate was also found to be negatively correlated with genome size^14^. We observed a higher correlation between genome size and flowering time of inbred lines evaluated under tropical growing conditions than under the temperate growing conditions (Fig. 1); the contribution of genome size to flowering time under tropical growing condition was also larger than that of the temperate conditions (Table 2). This result indicates that selection on genome size grown in tropical growing conditions will be more useful.

Using GWAS, we identified three genomic regions associated with flowering time, of which one region was mapped close to the knobs K8L1 and K8L2 on chromosome 8, which are annotated in maizeGDB. Two novel genomic regions were also identified in the centromere on chromosome 5 and chromosome 10. Previous studies have reported positive correlation between genome size in grass and the size of the centromere^43^ and also higher knob abundance in some centromeres as compared to other parts of the chromosomes^44, 45^. Therefore, it is not surprising that QTL for genome size are located in the centromere.

Our results showed a moderate correlation between 180-bp knob abundance and genome size. Given that there are 916 lines with whole genome sequences available in HapMap3, calculating knob abundance from these data might be used to obtain a quick estimate of genome size. Based on correlation between genome size and flowering time, selection for breeding materials with earlier flowering times may be facilitated by considering genome size and haplotypes consisting of favorable alleles associated with flowering time.

## Methods

### Quantification of maize genomic DNA

Maize inbred lines used for genomic DNA quantification (Table S4) were grown in a greenhouse under 28 °C/25 °C day/night and 16 h light/8 h dark. Four plants of each inbred line were grown per pot measuring 20-cm in diameter, 15-cm in height, and these pots were filled with 1:1 nutrient soil and vermiculite. Plants were watered with distilled water every two days. Three out of four plants of each maize inbred line were sent to PLANT CYTOMETRY SERVICES in the Netherlands for determination of absolute DNA content of plant nuclei (C-value). For each inbred line, DNA content was measured from three biological replicates and two technical replicates using flow cytometry as described in Enke *et al*.^46^. Genome size was estimated in picograms (pg) per 2 C nucleus using the nuclear DNA content of *Vinca major*, which is an evergreen plant with a relatively small DNA content (4.20 pg/2 C) compared to that of *Zea mays*, as an internal standard. Nuclear DNA content for the unknown samples (maize inbred lines) was calculated using the following formula^47^:

$$\mathrm{Sample}\,\,2{\rm{C}}\,\mathrm{value}(\mathrm{DNA}\,{\rm{pg}}\,{\rm{or}}\,\mathrm{Mbp})={\rm{Reference}}\,2{\rm{C}}\,{\rm{value}}\times \frac{\mathrm{Sample}\,2{\rm{C}}\,{\rm{mean}}\,{\rm{peak}}\,{\rm{position}}}{{\rm{Reference}}\,2{\rm{C}}\,{\rm{mean}}\,{\rm{peak}}\,{\rm{position}}}$$

For quality control, each measurement included more than 10,000 events, and the CV for most samples should be < 5–6%^23, 47^. For each maize inbred line, the average of six readings (three biological replicates and two technical replicates) was used to estimate the final genome size. Outliers in genome size were replaced as missing data. The R package was used to analyze the genome size data (http://www.r-project.org).

### Resequencing of maize lines

Using CTAB method^48^, genomic DNA was extracted from a single seedling from each of the 70 maize inbred lines grown under greenhouse conditions as described above for quantification of genomic DNA (Table S2). A library with insert size ranging between 400–600 bp was constructed from DNA extracted from each maize inbred line as described in Quail *et al*.^49^. Paired-end sequencing of each library was carried out on the HiSeq 2000 platform. We sequenced 90 bp from each paired end, and 6.0 Tb raw sequence data for the maize inbreds was obtained. Trimmomatic (version 0.30) was used to trim adapters in the raw reads^50^.

### Calculation of knob RPKM

The 180-bp knob sequences of temperate and tropical maize lines^24^ were downloaded from NCBI, and a 180-bp knob database was made using the protocol described by Tenaillon *et al*.^23^. SSAHA2 (version 2.5.5) was used to map genomic data of our 70 maize inbred lines to the 180-bp knob database with parameters -kmer 13 -skip 1 -identity 80 -memory 4000 -best 1 -score 12 -cmatch 9 -ckmer 6. Alignments < 30 bp were filtered out using kentUtils (http://github.com/ENCODE-DCC/kentUtils), and each alignment was considered a “hit”. The total number of knob hits was recorded for each inbred line. The 180-bp knob abundance indicated by RPKM^51^ was calculated following Tenaillon *et al*.^23^ as RPKM_i_ = *H*_*i*_/(*L*_*i*_ × *M* × 10^−6^), where *M* is the number of reads mapped against the 180-bp knob database, *H*_*i*_ is total the number of reads that map to the* i* ^*th*^ knob and *L*_*i*_ is the length of the knob in kilobases.

### Read mapping and assembly of 180-bp knobs

We selected 16 maize lines (Table S3) that had large differences in genome size to determine if 180-bp knobs are conserved or polymorphic. Reads for each of the 16 resequenced maize inbred lines were separately mapped to 180-bp knob sequences using SSAHA2 after excluding alignments < 30 bp were filtered out. SOAP de novo (version 2) was used to assemble the mapped reads into 180-bp knob sequences. Each assembled knob sequence was named based on sequence number, inbred line, and length of the assembled knob sequence. For example, the name 1833CNA004.180 indicates that this is the 1833rd sequence of the inbred line CNA004 and that the assembled knob sequence is 180-bp (Table S3). Reads of the resequenced maize lines were further mapped to the assembled knob sequences of 70 maize inbred lines by SOAP (version 2.21) with parameters -v 2 -p 20 -r 2. The top ten abundant sequences were aligned with DNAMAN 7.0.

### Phenotyping and analysis for flowering time

A total of 159 maize inbred lines that consisted of 74 tropical/subtropical and 85 temperate inbred lines (Table S4) were evaluated for flowering time. The 74 tropical/subtropical maize inbred lines were grown in a tropical region between October 2014 and May 2015, (Hainan-China, 18.1°N, 109.3°E) and in a temperate region between May and September 2013 (Beijing-China, 40.1°N, 116.6°E). The 85 temperate maize inbred lines were evaluated in a tropical region (Hainan-China, 18.1°N, 109.3°E) between October 2014 and May 2015 and in a temperate region between May and September 2014 (38.56°N, 100.27°E, Gansu-China), with two replicates in the same plot. All lines were planted using an incompletely randomized block design. Each plot was four meters long with 60 cm between rows and 25 cm between plants. The plots were single-row in the tropical region and the Gansu temperate region, and double-row in the Beijing temperate region. Flowering time was calculated as the number of days from planting until half of the plants in a row flowered. DTA and DTS were recorded for each inbred line. For the 85 temperate maize inbred lines evaluated in Gansu, DTA and DTS were averaged for flowering time analysis (For convenience, Gansu is labeled as Beijing). Photoperiod response of day to anthesis (PRDTA) or photoperiod response of day to silking (PRDTS) were calculated as the difference between DTA or DTS under long- and short-day conditions, respectively. Outliers in flowering time were replaced as missing data. Shapiro-Wilk test was used to check if flowering time data fit a normal distribution. Spearman correlation coefficients were computed to assess the relationship between flowering time and genome size (Fig. 3a). Population structure and kinship were introduced to the linear model to correct for population structure as described by Tenaillon *et al*.^14^. Data were analyzed using the R package (https://www.r-project.org/).

### GWAS and the genotype data used

A total of 175 maize inbred lines that includes 93 tropical/subtropical and 82 temperate inbreds were used for GWAS (Table S5). DNA was extracted from each inbred line using the CTAB protocol following Murray *et al*.^48^. The DNA samples were genotyped using a newly developed 55 K Array invented by our lab^40^ at Capitalbio Technology Beijing. Genome size was used to predict positions associated with it. The 175 inbred lines for genotype were grown under the same environment conditions and the same methods as those inbred lines grown for genome size quantification. GWAS was conducted using mixed linear model (MLM) implemented in TASSEL software (version 5.0)^52^ using the following input files: (i) the first three principal components from principal component analysis (PCA) as covariates to account for population structure; (ii) the identity by state (IBS) based on kinship matrix to account for population relatedness; (iii) the genome size determined by flow cytometry; (iv) the 38,765 genotype data after filtering SNPs with minor allele frequency less than 0.05. Manhattan plots were obtained from genome-wide *P* using a graphical tool for SNP effect viewing and graphing (SNPevg)^53^.

## Electronic supplementary material

Supplementary Information

Table S1

Table S2

Table S3

Table S4

Table S5

Table S6
