# Supplementary material for: Maize (Zea mays L.) genome size indicated by 180-bp knob abundance is associated with flowering time
Source: Sci Rep. 2017 Jul 20;7:5954. doi: 10.1038/s41598-017-06153-8 (PMC5519714; doi:10.1038/s41598-017-06153-8)
Supplement: Supplementary file 1 — Supplementary Information [file 41598_2017_6153_MOESM1_ESM.pdf]

Supplementary Information

Maize (*Zea mays* L.) genome size indicated by 180-bp knob abundance is associated with flowering time

Yinqiao Jian[1], Cheng Xu[1], Zifeng Guo[1], Shanhong Wang[1], Yunbi Xu[1, 2] & Cheng Zou[1]*

[1]Institute of Crop Science, National Key Facility of Crop Gene Resources and Genetic Improvement, Chinese Academy of Agricultural Sciences, Beijing 100081, China

[2]International Maize and Wheat Improvement Center (CIMMYT), El Batán 56130, Texcoco, Mexico

*Correspondence and requests for materials should be addressed to C.Z. (zoucheng@caas.cn)

Supplementary Figure S1. Flowering time distribution

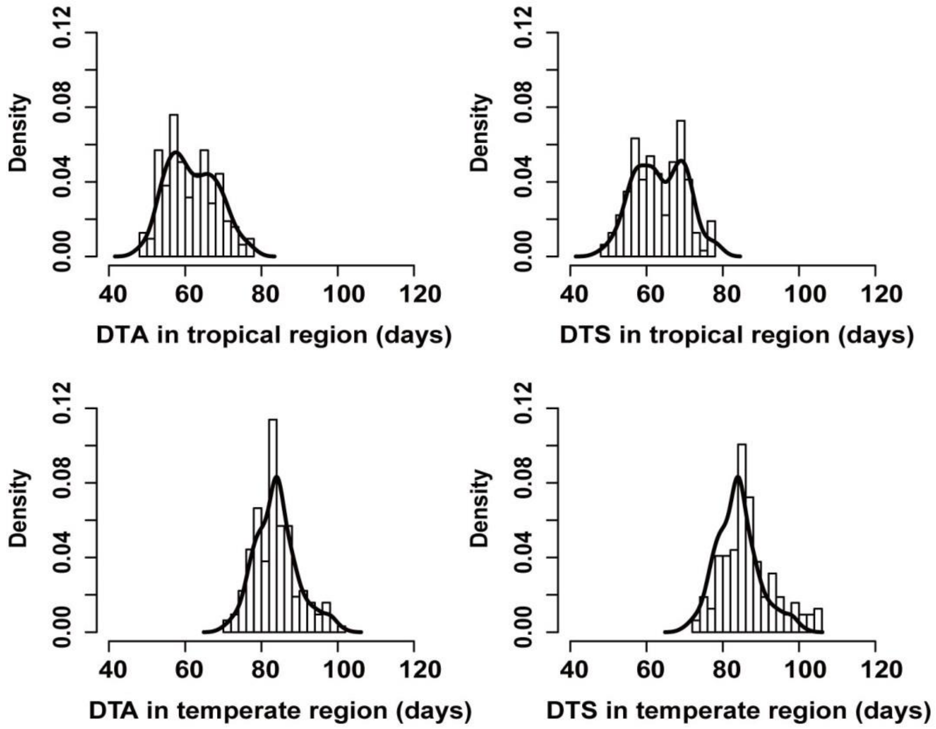

Supplementary **Figure 1.** Distributions of DTA and DTS in tropical and temperate maize inbred lines in tropical and temperate regions. Shapiro-tests indicate that DTA and DTS in both locations do not follow a normal distribution (P < 0.05). P are 0.0071 for DTA in the tropical region, 0.0083 for DTS in the tropical region, 0.0046 for DTA in the temperate region and 0.0002 for DTS in the temperate region.

DTS: days to silking; DTA: days to anthesis

Supplementary Table list

Table S1. Genome sizes for tropical and temperate inbred maize lines

Table S2. Genome size and 180-bp knob abundance

Table S3. Differences in the number of 180-bp knobs among different maize lines

Table S4. Flowering time for tropical and temperate inbred maize lines

Table S5. Materials for GWAS

Table S6. 38765 genotype for GWAS
